# The Prognostic Value of GNG7 in Colorectal Cancer and Its Relationship With Immune Infiltration

**DOI:** 10.3389/fgene.2022.833013

**Published:** 2022-02-23

**Authors:** Can Fang, Rulei Zhong, Chenyang Qiu, Bing-bing Zou

**Affiliations:** Department of General Surgery, First Affiliated Hospital of Anhui Medical University, Hefei, China

**Keywords:** GNG7(G Protein Subunit Gamma 7), prognosis, CRC, colorecta cancer, immune infiltration

## Abstract

**Background**: G Protein Subunit Gamma 7 (GNG7) is an important gene that regulates cell proliferation and induces apoptosis. However, the correlation between GNG7 expression and immune infiltration as well as patient prognosis of colorectal cancer (CRC) remains unclear.

**Methods**: The GNG7 expression differences between tumor tissues and normal tissues were explored via the Oncomine database, Tumor Immune Estimation Resource (TIMER) site and UALCAN database. Then, the influence of GNG7 on clinical prognosis were evaluated, using the PrognoScan database. In addition, the relationship between GNG7 and tumor-related immune infiltration as well as gene marker sets of immune infiltration was investigated via TIMER, TISIDB and GEPIA.

**Results**: We found that GNG7 expression was down-regulated in multiple malignant tumors including colorectal cancer (CRC) and the GNG7 expression was associated with tumor stage, histology subtype, lymph node metastasis and poor prognosis in colorectal cancer (CRC). In addition, the expression of GNG7 was significantly associated with infiltration level of multiple immune cells, immunomodulatory factors as well as part of the immune cell markers.

**Conclusion**: GNG7 displays validated prognostic value in CRC and was associated with its immune cell infiltration and immunoregulation. These results suggest that GNG7 is a potential prognostic marker and is associated with tumor immune infiltration, thus providing a new perspective for the immunotherapy of CRC.

## Introduction

In 2020, there were approximately more than 1.9 million new cases of colorectal cancer (CRC) and 935,000 deaths from colorectal cancer globally. Overall, colorectal cancer accounting for the third most common cancer in the world, but ranks second in terms of mortality (Sung et al., 2021). At present, surgery is still the cornerstone of the treatment of CRC, but its shortcomings are also obvious. For example, the choice of surgical methods and the effectiveness of surgical treatment are largely affected by the depth of tumor invasion and anatomical location (Brenner et al., 2014). Although significant progress has been made in the screening, diagnosis, surgery and chemotherapy for patients with colorectal cancer in recent decades, the patients’ clinical benefits acquired from traditional treatment approaches are limited and the patients’ prognosis still needs to be improved. It is reported that about 25% of patients with CRC have metastases at the time of diagnosis, and approximately 50% of them will eventually metastasize in their lifetime (Vatandoust et al., 2015).

Multiple immune cells are vital components of the tumor microenvironment (TME) and deeply influence tumor occurrence and development (Hinshaw and Shevde 2019). Nowadays, immunotherapy has achieved satisfactory effects in dealing with some malignant tumors such as small cell lung cancer, renal cancer and breast cancer (Motzer et al., 2015; Gandhi et al., 2018; Schmid et al., 2018). However, there are still many unknown areas worth exploring in the field of immunotherapy for colorectal cancer. For example, the application of ICIS (immune checkpoint inhibitors) in CRC has epoch-making significance for the immunotherapy of CRC. Nevertheless, less than 15% of colorectal cancer patients exhibit this predictive biomarker, so the vast majority of the remaining CRC patients currently have no opportunity to extend survival with immunotherapy (Lichtenstern et al., 2020). Therefore, to determine immunotherapy targets by studying the influencing factors of tumor-related immune infiltration will enrich the immunotherapy approaches of colorectal cancer and help improve the clinical outcome of cancer patients.

GNG7 belongs to the large guanine nucleotide binding protein γ family and is located on chromosome 19 (Shibata et al., 1998). Related genes of the G protein family are involved in a variety of transmembrane signaling transduction pathways. In addition, studies have shown that GNG7 gene may also be associated with transmembrane signaling pathways and is involved in cell contact-induced growth arrest, thereby inhibiting uncontrolled cell proliferation in multicellular organisms (Hartmann et al., 2012). GNG7 is ubiquitously expressed in multiple tissues but is down-regulated in various cancers (Liu et al., 2016). Several studies have shown that its expression is decreased in pancreatic cancer (Liu et al., 2019), esophagus cancer, gastrointestinal tract cancer (Shibata et al., 1999), and clear cell renal cell carcinoma (ccRCC) (Xu et al., 2019). Combined with the research conclusions listed above, it can therefore be regarded as a potential suppressor of malignant tumor.

The expression profiles and prognostic value of GNG7 in CRC are still unclear. In addition, the association between GNG7 and immune infiltration in CRC still remains largely unexplored. Elucidating these issues will pave the way for the development of new treatments for CRC.

In this study, we analyzed the expression of GNG7 in various cancers through Oncomine, UALCAN, and TIMER databases. Furthermore, the prognostic significance of GNG7 in different tumors was investigated by the PrognoScan web servers. Moreover, we investigated the correlation between GNG7 expression and immune infiltration as well as immunoregulation level in colorectal cancer via TIMER, TISIDB, and GEPIA databases. Our findings revealed the prognostic value of GNG7 in CRC, and provide new insights into the correlation and potential mechanism between GNG7 expression and CRC immune infiltration.

## Materials and Methods

### Tissue Samples

We collected 20 samples of colorectal cancer and para-cancerous tissues as well as related clinical information from the First Affiliated Hospital of Anhui Medical University (Supplementary Table S2). None of the subjects received radiotherapy or chemotherapy before surgery. All patients’ data were obtained with informed consent, and this study was approved by the Clinical Research Ethics Committees. (Tissue specimen collection time is from 10 June 2021 to 20 July 2021).

### Quantitative Real-Time PCR

TRIzol reagent (Invitrogen, Carlsbad, CA, United States) was used for total RNA extraction from tissues. The assay was conducted in triplicate and performed using 2× TB Green Premix Ex Taq (Takara) in a Bio-Rad detection system. All reactions were repeated. Relative gene expression was calculated by the 2–ΔΔCt method. GAPDH served as the internal reference. The primer sequences were as follows (“F” represents “forward”; “R”represents“reverse”). GAPDH: 5′-AGAAGGCTGGGGCTCATTTG-3′(F), 5′AGGGGCCATCCACAGTCTTC-3′(R); GNG7:5′-CCCGAGCGCAGGGAGT-3′(F), 5′-CGC​TCA​ATC​CCG​GCT​TCT​AT-3′(R).

### Oncomine Database Analysis

The oncomine database (http://www.oncomine.org/) was designed for the mining and analysis of tumor-related data (Rhodes et al., 2007). We employed it to analyze the expression differences of GNG7 in tumor tissues and adjacent normal tissues on it. The threshold was defined as a *p*-value < 0.01, a fold change>2, and a gene ranking of 10%.

### PrognoScan Database Analysis

PrognoScan (http://www.prognoscan.org/) database can be used to analyze the relationship between gene expression and different prognostic outcomes (including OS, DFS, etc.) of patients (Mizuno et al., 2009). We analyzed the correlation between GNG7 expression and survival outcomes in different types of cancers and generated corresponding survival curves.

### UALCAN Database Analysis

UALCAN (http://ualcan.path.uab.edu/index.html) is a practical online analysis database with multiple function modules, which is widely used in TCGA data analysis (Chandrashekar et al., 2017). We used UALCAN to analyze the mRNA and protein expression levels of GNG7 in tumor and adjacent normal tissues, as well as its relationship with clinicopathological features.

### Tumor Immune Estimation Resource Database Analysis

Tumor Immune Estimation Resource (https://cistrome.shinyapps.io/timer/) (TIMER) platform is a comprehensive resource database which can be used to analyze differential expression of genes and the relationship between specific gene expression and the abundance of immune cell infiltration in various cancers, and the abundance of tumor-infiltrating immune cell (TIIC) is calculated use specific algorithms (Li et al., 2017). We used TIMER to explore the GNG7 expression in different types of cancers and the correlation between GNG7 expression and the infiltration abundance of six kinds of immune cells (TIICs, including B cells, CD4 + T cells, CD8 + T cells, neutrophils, macrophages, and dendritic cells (DCs)). In addition, the relationship between GNG7 expression and immune cell marker genes was further analyzed, and the results were presented in the form of a scatter plot with the Spearman correlation coefficient and *p*-value. Gene expression levels were shown as log2 TPM.

### Gene Expression Profiling Interactive Analysis Database Analysis

The Gene Expression Profiling Interactive Analysis (http://gepia.cancer-pku.cn/index.html) (GEPIA) database integrates wide bioinformatics data, which can be used for a variety of bioinformatics research including genetic difference analysis, prognosis analysis and gene correlation analysis (Tang et al., 2017). The correlation between GNG7 expression and specific immune cell marker genes identified in TIMER database previously was further proved in GEPIA.

### TISIDB Database Analysis

TISIDB database (https://cis.hku.hk/TISIDB) is a web portal for conducting tumor and immune system interactions analysis that integrates multiple heterogeneous data types (Ru et al., 2019). We used this database to help investigate the correlation between GNG7 expression and tumor-infiltrating lymphocytes as well as immunomodulators.

### Schematic Diagram and Flow Chart

**FIGURE 1:**
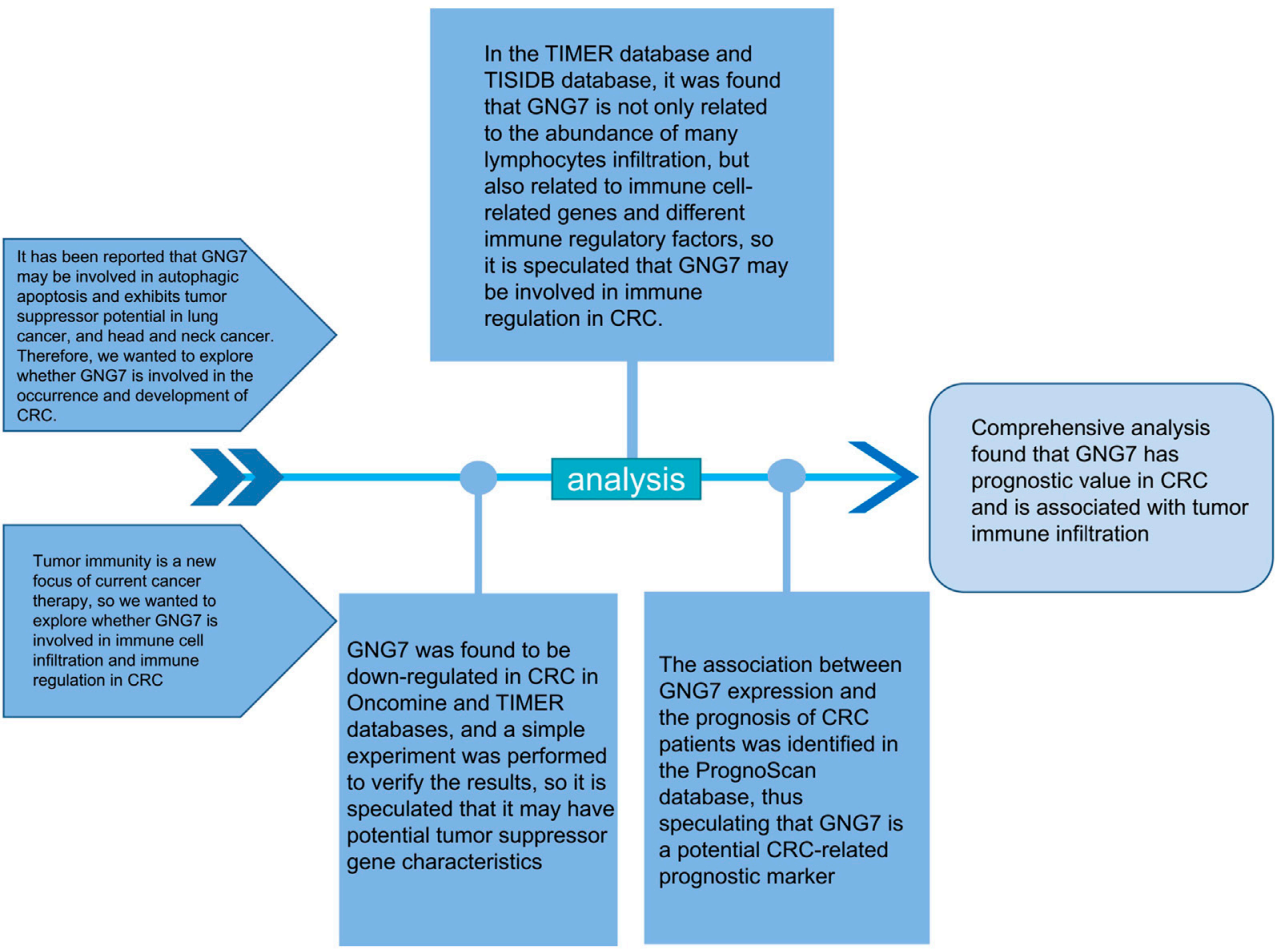
Illustration of the main principle of this study.

**FIGURE 2:**
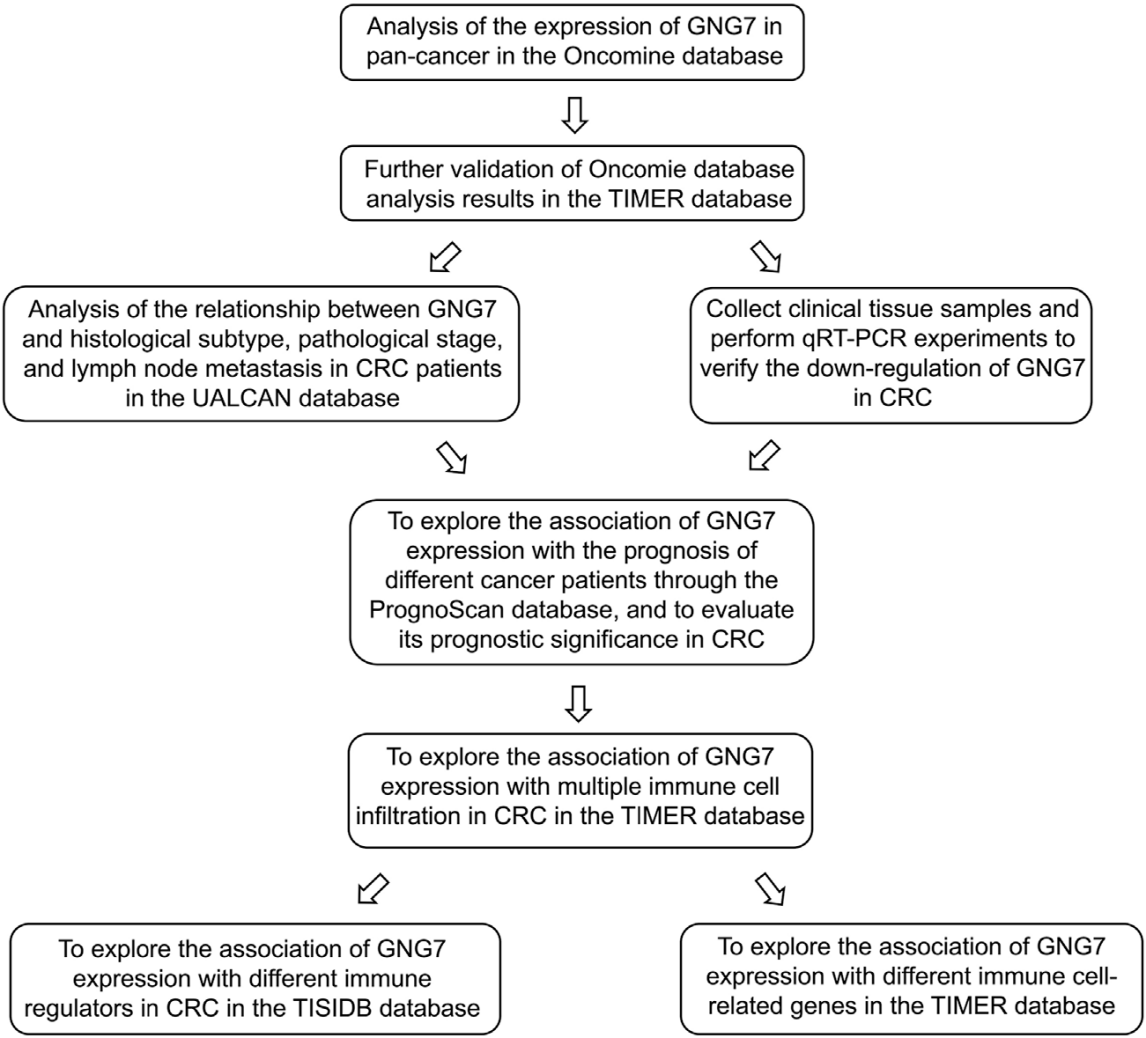
The basic analysis process of this study.

Well-designed diagrams help readers better understand the researcher’s research content (Behzadi and Gajdács 2021). For this reason, we present the schematic diagram (Figure 1) and technical flow chart (Figure 2) of the entire research process in the article for easy reference.

### Statistical Analysis

Survival curves were generated from the PrognoScan and the results displayed with HR and P or Cox *p*-value from a log-rank test. The results generated from Oncomine were displayed with *p*-value, fold changes and ranks. The correlation of gene expression with the lymphocytes, immunomodulators and gene markers of tumor-infiltrating immune cells was evaluated by Spearman’s correlation coefficient and statistical significance. *p*-value <0.05 is considered statistically significant.

## Results

### The mRNA Expression Levels of GNG7 in Different Types of Human Cancers

**FIGURE 3:**
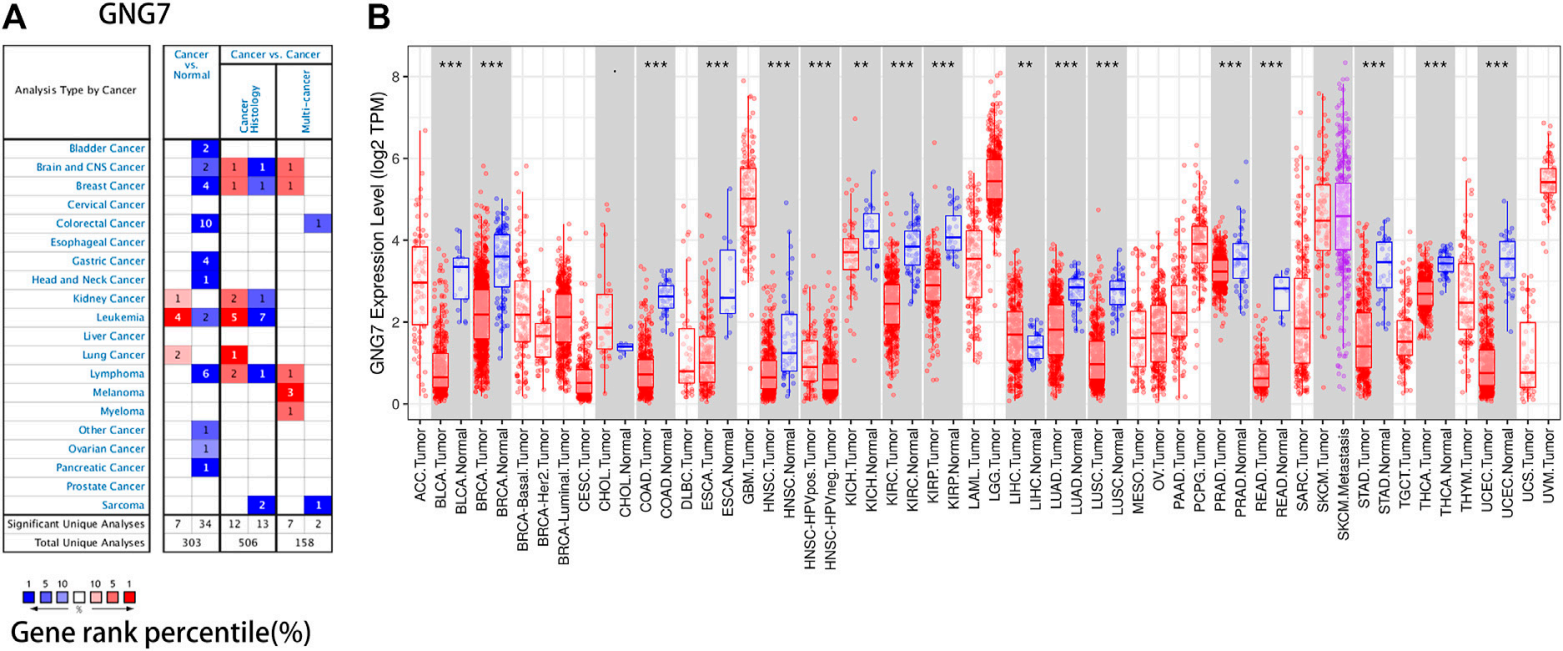
GNG7 mRNA expression in a variety of human cancers. **(A)** GNG7 expression was downregulated or upregulated in diverse cancers in the Oncomine database. **(B)** Human GNG7 expression in multiple types of cancer was determined using the TIMER database (*p < 0.05, **p < 0.01, ***p < 0.001).

The differences of GNG7 expression between tumor and normal tissues were analyzed in Oncomine database. It was observed that the GNG7 expression was up-regulated in kidney cancer, leukemia and lung cancer compared with the normal tissues, while it was down-regulated significantly in some datasets of bladder cancer, brain and CNS cancer, breast cancer, colorectal cancer, gastric cancer, head and neck cancer, leukemia, lymphoma, ovarian cancer and pancreatic cancer (Figure 3A, Supplementary Table S1).

**FIGURE 4:**
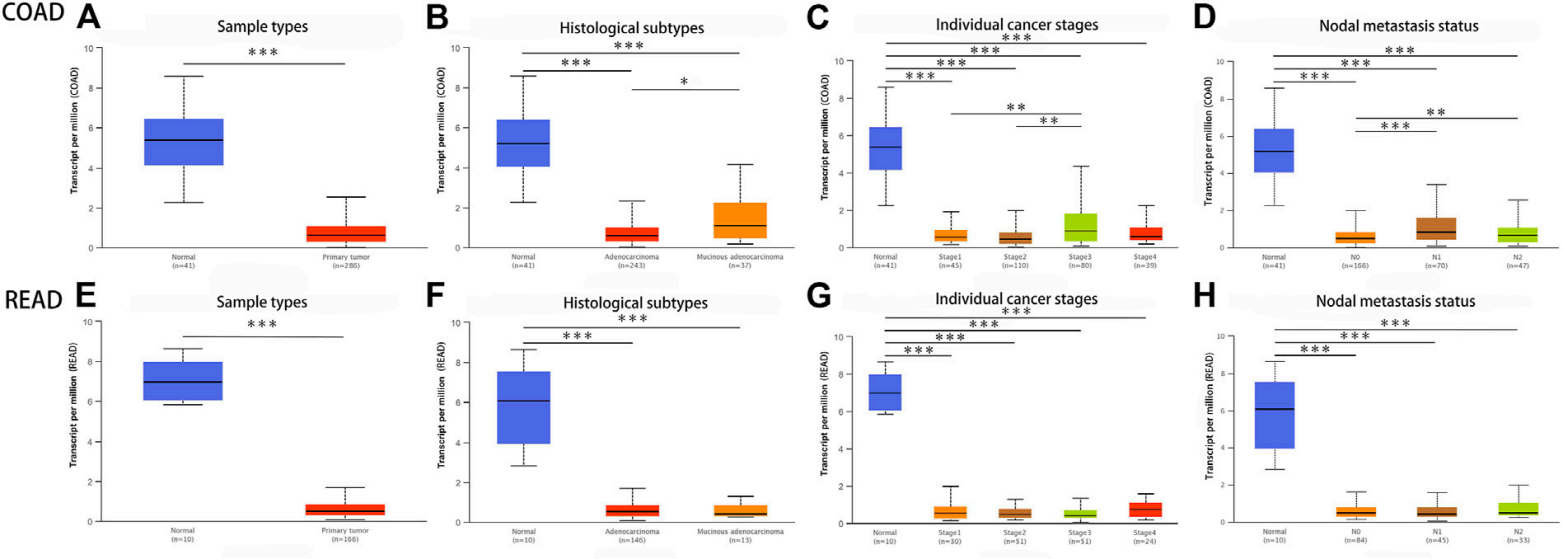
The relationship between GNG7 expression and individual clinical parameters of COAD and READ in UALCAN database. **(A)** Differential expression of GNG7 in COAD tissues. Association of GNG7 mRNA expression with different COAD patients’ individual cancer histologic subtypes **(B)**, tumor stages **(C)**, nodal metastasis **(D)**. **(E)** Differential expression of GNG7 in READ tissues. Association of GNG7 mRNA expression with different READ patients’ individual cancer histologic subtypes **(F)**, tumor stages **(G)**, nodal metastasis **(H)**.

To further verify the outcome from Oncomine database, we examined the GNG7 expression levels in multiple malignancies from TCGA database in TIMER. The differential expression between the tumor and adjacent normal tissues of GNG7 was shown in Figure 3B, and as we can see, GNG7 was down-regulated in various cancers, especially in colon cancer and rectal cancer, which was consistent with the results in Oncomine. In addition, we found that GNG7 expression was not only down-regulated in COAD and READ, but it was also associated with tumor histology, stage, lymph node metastasis according to the result in UALCAN (Figure 4).

**FIGURE 5:**
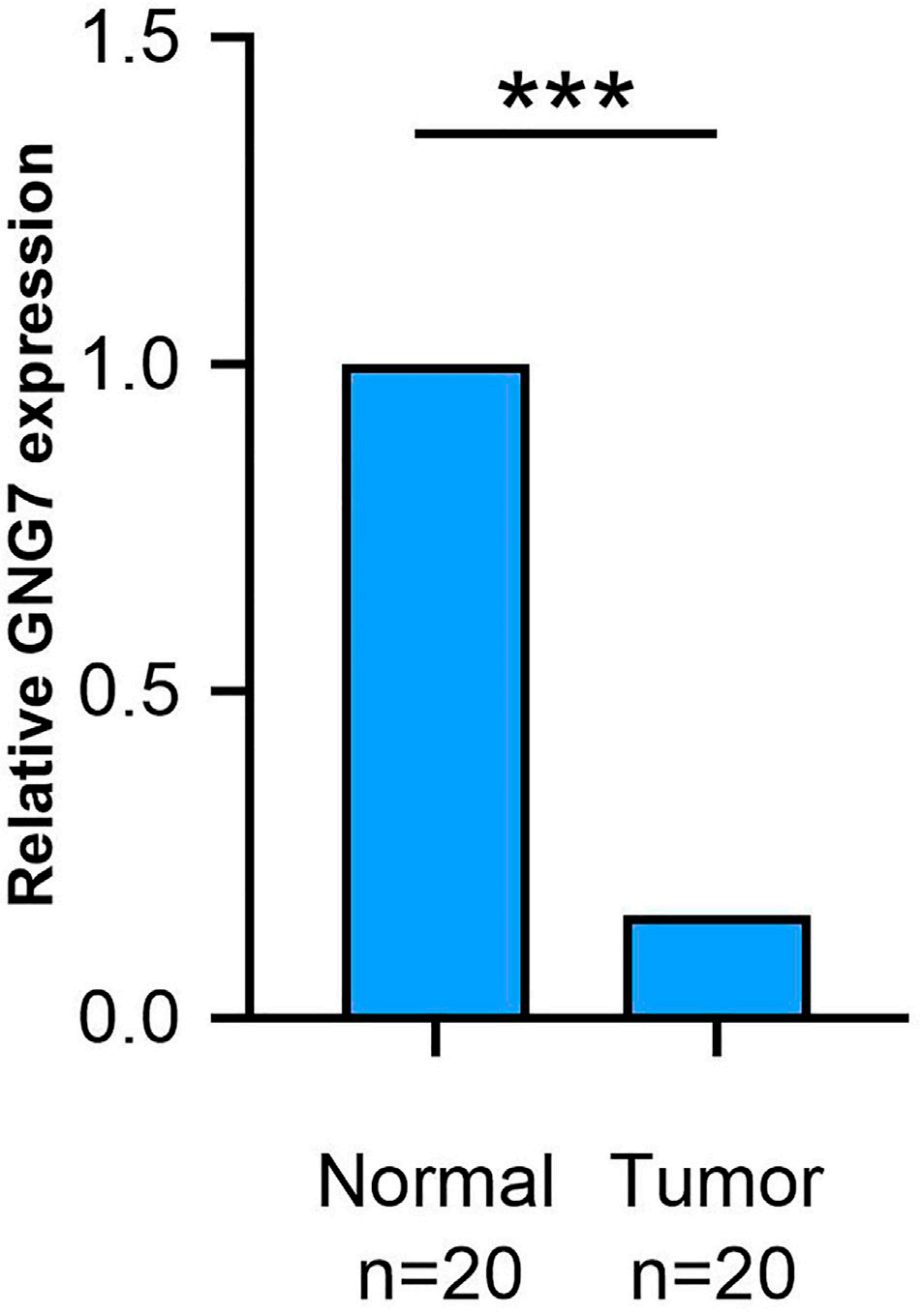
The results of RT-qPCR experiments are consistent with the analysis results of Oncomine and TIMER databases. The expression level of GNG7 mRNA in colorectal cancer tumor tissues is significantly down-regulated compared with normal para-cancerous tissues. ***p < 0.0001.

We used qRT-PCR to identify the expression of GNG7 in CRC tissues and para-cancerous tissues. The results are displayed in the form of a histogram. It is confirmed that the GNG7 mRNA level was decreased in CRC tissues than para-cancerous tissues (Figure 5, In addition, experimental related pictures are shown in Supplementary File S1). These results suggests that GNG7 was obviously lowly expressed in various cancers, especially in CRC.

### Prognostic Potential of GNG7 Across Different Cancers

**FIGURE 6:**
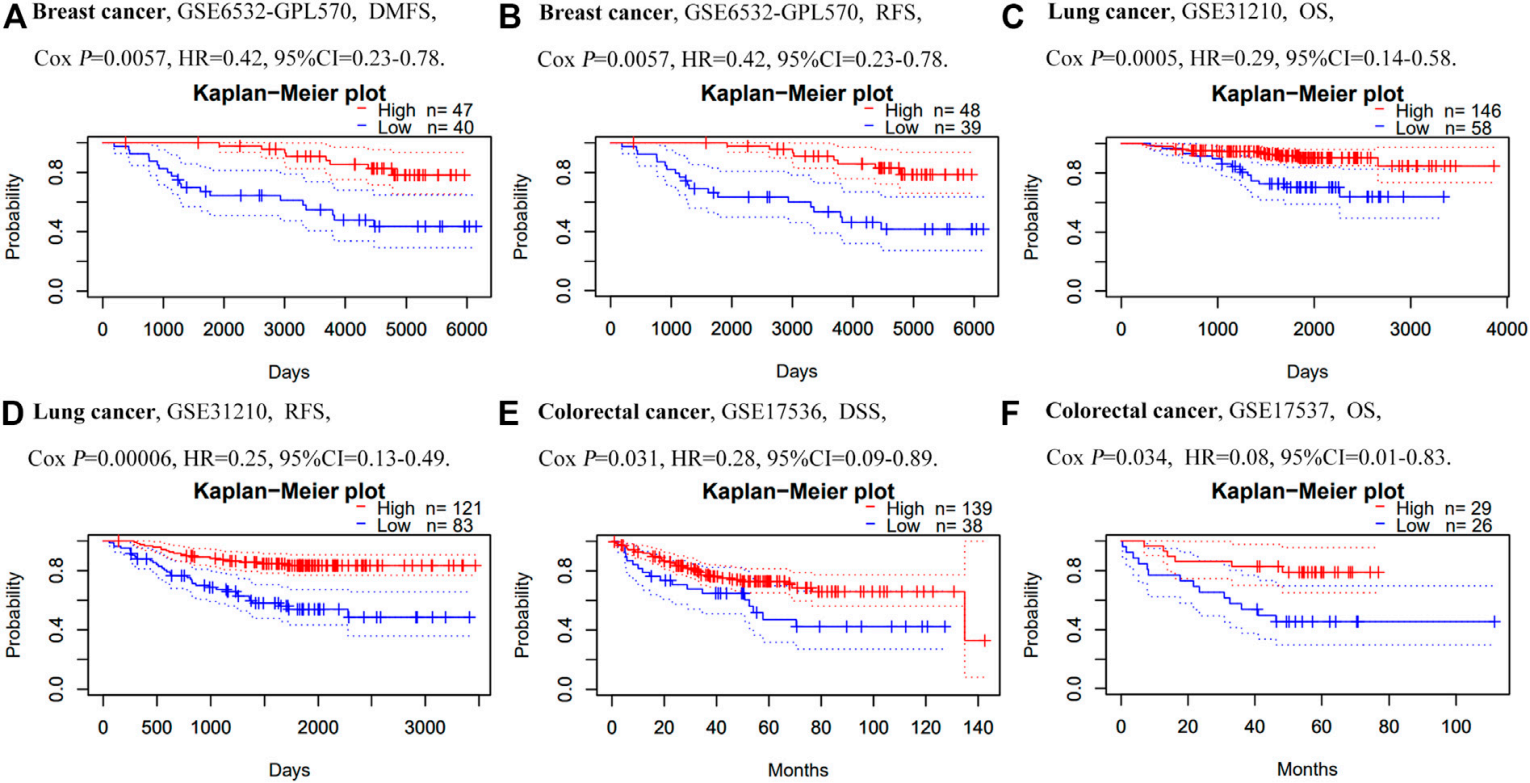
Association of GNG7 mRNA expression with prognosis in different human cancers in the PrognoScan. **(A–B)** Survival curves of DMFS and RFS in a breast cancer cohort (GSE6532-GPL570, *n* = 87). **(C–D)** Survival curves of OS and RFS in a lung cancer cohort (GSE31210, *n* = 204). **(E–F)** Survival curves of DSS and OS in two colorectal cancer cohorts [GSE17536 (*n* = 177) and GSE17537 (*n* = 55)].

In order to investigate the prognostic value of GNG7 expression in different types of cancers, we further analyzed the relationship between GNG7 expression and prognosis of cancer patients via PrognoScan database. The analysis uncovered that GNG7 expression was significantly related to the prognosis of eight types of tumors including lung cancer, breast cancer and colorectal cancer (Figure 6). For example, it can be observed that in the GSE6532-GPL570 cohort which included 87 breast cancer samples, the discrepancies of DMFS (Distant Metastasis Free Survival) and RFS (Relapse Free Survival) between two groups were significant. In the GSE31210 cohort which included 204 lung cancer samples, high GNG7 expression was associated with better OS (Overall Survival, *p* = 0.0005, HR = 0.29,95%CI 0.14–0.58) and RFS (*p* = 0.0002, HR = 0.25,95%CI 0.13–0.49). Notably, In the GSE17536 and GSE17537 cohorts, which included 177 and 55 colorectal cancer samples respectively, high GNG7 expression was associated with better OS (*p* = 0.034, HR = 0.08,95%CI 0.01–0.83) and DSS (Disease Specific Survival, *p* = 0.031, HR = 0.28,95%CI 0.09–0.89). Hence, these findings indicated that GNG7 can be regarded as a potential indicator for predicting the prognosis of certain types of cancer including CRC.

### GNG7 Expression is Correlated With Immune infiltration Level in Colorectal Cancer

**FIGURE 7:**
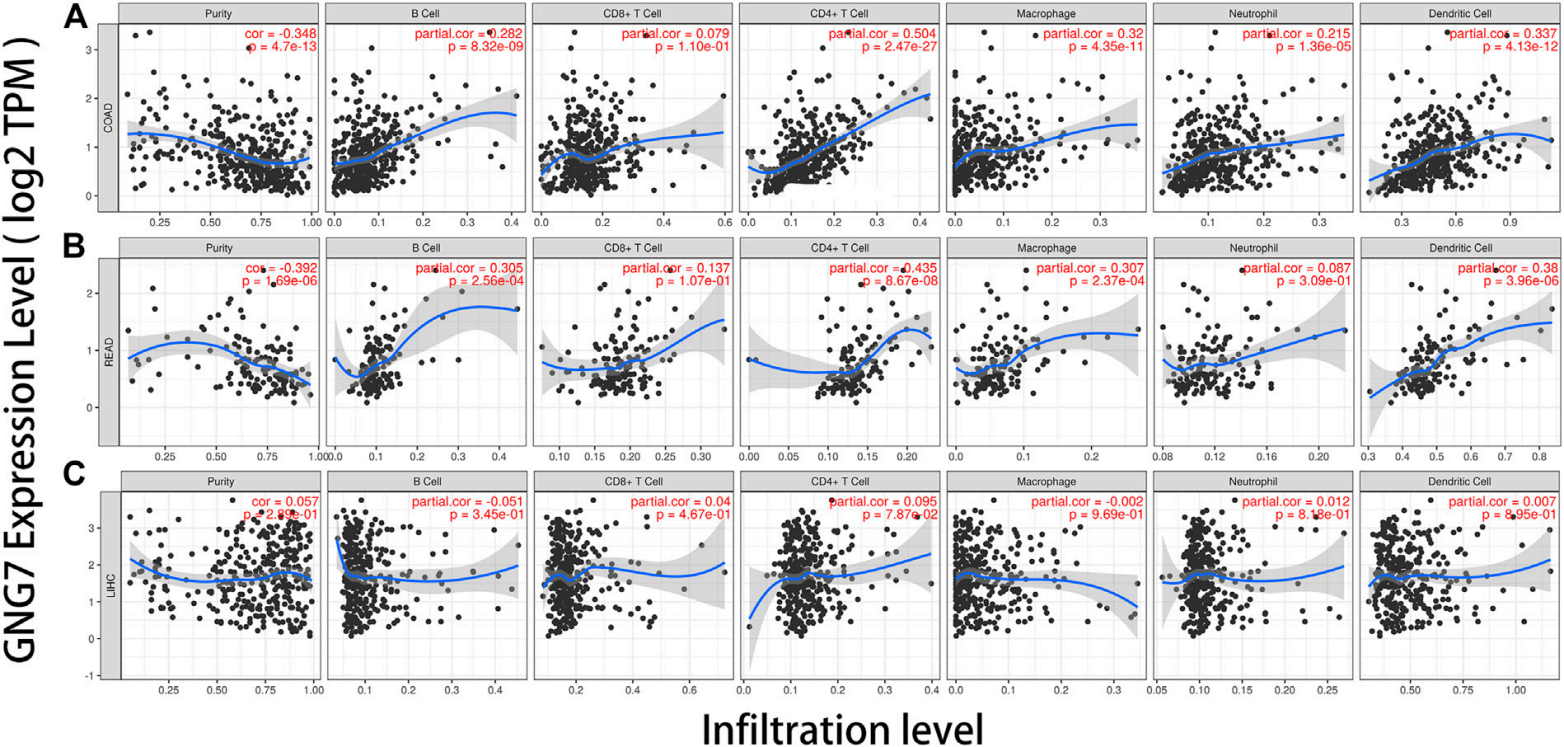
Correlation of GNG7 expression with immune infiltration level in COAD (colon adenocarcinoma), READ (rectum adenocarcinoma), and LIHC (liver hepatocellular carcinoma). **(A)** GNG7 expression is significantly negatively related to tumor purity and has significant positive correlations with infiltrating levels of B cells, CD4^+^ T cells, macrophages, neutrophils, and dendritic cells in COAD, other than CD8^+^ T cells. **(B)** GNG7 expression is significantly negatively related to tumor purity and has significant positive correlations with infiltrating levels of B cells, CD4^+^ T cells, macrophages, and dendritic cells in READ but no significant correlation with infiltrating level of CD8^+^ T cells and neutrophils. **(C)** GNG7 expression has no significant correlations with tumor purity and infiltrating levels of B cells, CD8^+^ T cells, CD4^+^ T cells, macrophages, neutrophils, and dendritic cells in LIHC.

The infiltration of tumor-related immune cells was regarded as an independent factor affecting the prognosis of patients with colorectal cancer (Ohtani 2007). Therefore, we explored the relationship between GNG7 expression and the abundance of immune infiltration in different types of tumors via TIMER database. Tumor purity is an important reference factor in analyzing the degree of tumor immune infiltration (Yoshihara et al., 2013). The results showed that the expression of GNG7 was significantly correlated with tumor purity in 21 types of cancer, including COAD and READ. In addition, the expression of GNG7 had significant correlations with the infiltration level of B cells in 18 tumors, CD8^+^ T cells in 17 tumors, CD4^+^ T cells in 27 tumors, macrophages in 22 tumors, neutrophils in 14 tumors, and dendritic cells in 21 tumors, respectively. Notably, we found that the GNG7 expression level was correlated with poor prognosis and high immune infiltration in COAD and READ. GNG7 expression level had significant positive correlations with infiltrating levels of B cells (*r* = 0.282, *p* = 8.32e-09), CD4^+^ T cells (*r* = 0.504, *p* = 2.47e-27), macrophages (*r* = 0.320, *p* = 4.35e-11), neutrophils (*r* = 0.215, *p* = 1.36e-05) and DCs (*r* = 0.337, *p* = 4.13e-12) in COAD (Figure 7A). Moreover, GNG7 expression levels were also positively associated with infiltration levels of B cells (*r* = 0.305, *p* = 2.56e-04), CD4^+^ T cells (*r* = 0.435, *p* = 8.67e-08), macrophages (*r* = 0.307, *p* = 2.37e-04) and DCs (*r* = 0.380, *p* = 3.96e-06) in READ (Figure 7B). In contrast, GNG7 expression had no significant correlation with tumor purity and infiltrating levels of B cells, CD8^+^ T cells, macrophages, neutrophils, or dendritic cells in LIHC (Figure 7C).

### The Function of GNG7 in Regulating Immune Molecules

**FIGURE 8:**
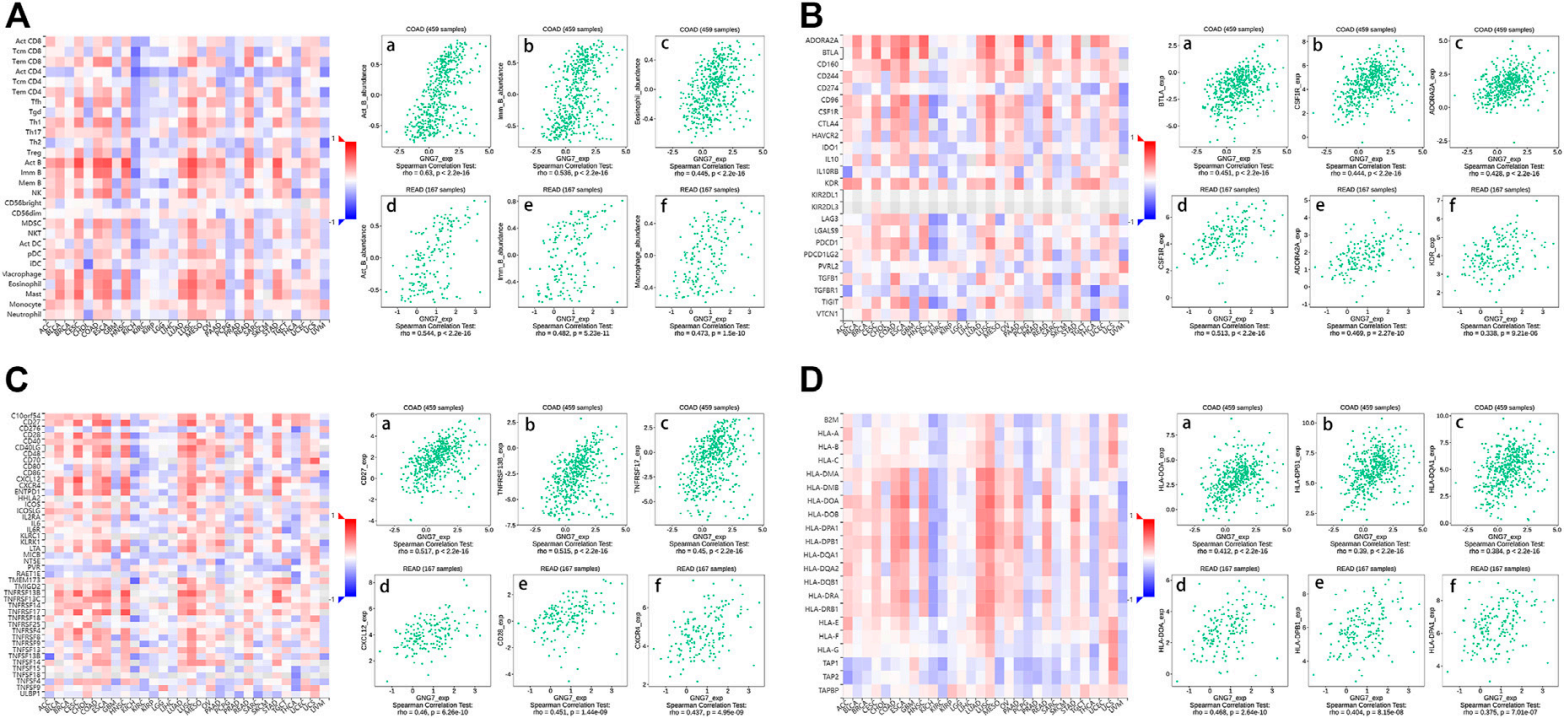
Spearman’s correlation of GNG7 with lymphocytes and immunomodulators in TISIDB database. **(A)** Relationship between the abundance of TILs and GNG7 expression in different human cancers. Top 3 TILs displaying the greatest correlation with GNG7 expression in COAD **(A–C)** and READ **(D–F)**. **(B)** Relationship between the abundance of immunoinhibitors and GNG7 expression in different human cancers. Top 3 immunoinhibitors displaying the greatest correlation with GNG7 expression in COAD **(A–C)** and READ **(D–F)**. **(C)** Relationship between the abundance of immunostimulators and GNG7 expression in different human cancers. Top 3 immunostimulators displaying the greatest correlation with GNG7 expression in COAD **(A–C)** and READ **(D–F)**. **(D)** Relationship between the abundance of MHC molecules and GNG7 expression in different human cancers. Top 3 MHC molecules displaying the greatest correlation with GNG7 expression in COAD **(A–C)** and READ **(D–F)**.

Then, in order to study the regulation mechanism of GNG7 on immune molecules in CRC, we further conducted an integrated analysis on the correlation between GNG7 expression and 28 tumor immune infiltrating cell subtypes as well as three subtypes of immunomodulators in TISIDB database. The result showed that GNG7 was associated with twenty-four immune cell subtypes in COAD and READ (Table 1). Notably, Activated B cell (rho = 0.63, *p* < 2.2e-16), Immature B cell (rho = 0.536, *p* < 2.2e-16) and Eosinophil (rho = 0.445, *p* < 2.2e-16) occupied the top three TILs in terms of the strength of the correlation with GNG7 expression in COAD (Figures 8A–C), while the top three TILs with the strongest correlation with GNG7 expression in READ were Activated B cell (rho = 0.544, *p* < 2.2e-16), Immature B cell (rho = 0.482, *p* = 5.23e-11) and Macrophage (rho = 0.473, *p* < 1.5e-10) (Figure 8D–F). Immunoinhibitors, immunostimulators and major histocompatibility complex (MHC) molecules comprised all immunomodulators (Gou et al., 2019). The correlation heat maps of GNG7 expression with immunoinhibitors, immunostimulators and major histocompatibility complex (MHC) molecules in different cancers were displayed respectively in (Figures 8B–D). In addition, the correlation scatter plots of the three immunomodulators which had the strongest correlation with GNG7 expression in COAD and READ were displayed near the related heat maps. The specific coefficients and *p* value were also shown in Supplementary Table S3. These findings provided compelling evidence for demonstrating that GNG7 plays a specific role in immune infiltration in colorectal cancer.

**TABLE 1:** The correlation between GNG7 expression and tumor lymphocyte infiltration in COAD, READ and LIHC(TISIDB).

Lymphocyte subtypes	COAD		READ		LIHC	
	Cor	p	Cor	p	Cor	p
Activated CD8 T cell (Act _CD8)	0.136	0.0035	0.142	0.0664	0.016	0.751
Central memory CD8 T cell (Tcm _CD8)	0.186	6.22e-05	0.378	5.91e-07	−0.004	0.932
Effector memory CD8 T cell (Tem _CD8)	0.433	<2.2e-16	0.471	1.92e-10	0.05	0.337
Activated CD4 T cell (Act _CD4)	0.007	0.873	−0.099	0.201	−0.389	5.46e−15
Central memory CD4T cell (Tcm _CD4)	0.219	2.2e−06	0.316	3.41e−05	−0.177	0.000619
Effector memory CD4 T cell (Tem _CD4)	0.222	1.66e−06	0.259	0.00074	−0.197	0.000136
T follicular helper cell (Tfh)	0.37	2.13e−16	0.441	3.74e−09	−0.131	0.0113
Gamma delta T cell (Tgd)	0.224	1.34e−06	0.275	0.000333	−0.117	0.0241
Type 1 T helper cell (^1^Th)	0.419	<2.2e−16	0.447	2.07e−09	0.001	0.99
Type 17 T helper cell (Th17)	0.156	0.00083	0.105	0.178	−0.203	8.2e−05
Type 2 T helper cell (Th2)	0.263	1.19e−08	0.23	0.00288	−0.229	8.68e−06
Regulatory T cell (Treg)	0.359	2.55e−15	0.434	6.48e−09	−0.123	0.0172
Activated B cell (Act _B)	0.63	<2.2e−16	0.544	<2.2e−16	0.037	0.482
Immature B cell (Imm _B)	0.536	<2.2e−16	0.482	5.23e−11	−0.12	0.0205
Memory B cell (Mem _B)	0.155	0.000859	0.134	0.0839	−0.127	0.014
natural killer cell (NK)	0.411	<2.2e−16	0.47	2.1e−10	0.065	0.211
CD56bright natural killer cell (CD56bright)	0.051	0.271	0.016	0.837	0.071	0.173
CD56dim natural killer cell (CD56dim)	0.049	0.298	0.118	0.13	0.062	0.23
Myeloid derived suppressor cell (MDSC)	0.427	<2.2e−16	0.441	3.74e−09	−0.14	0.00686
Natural killer T cell (NKT)	0.337	1.54e−13	0.379	5.36e−07	−0.13	0.0121
Activated dendritic cell (Act _DC)	0.137	0.00319	0.2	0.00959	−0.188	0.00026
Plasmacytoid dendritic cell (pDC)	0.263	1.28e−08	0.355	2.84e−06	−0.097	0.0615
Immature dendritic cell (iDC)	0.14	0.00262	0.182	0.0187	−0.139	0.00711
Macrophage (Macrophage)	0.433	<2.2e−16	0.473	1.5e−10	−0.062	0.229
Eosinophil (Eosinophil)	0.445	<2.2e−16	0.321	2.61e−05	0.076	0.142
Mast (Mast)	0.436	<2.2e−16	0.465	3.61e−10	−0.085	0.0994
Monocyte (Monocyte)	0.101	0.0313	0.195	0.0115	0.116	0.0254
Neutrophil (Neutrophil)	0.203	1.24e−05	0.333	1.2e−05	−0.188	0.00027

### Correlation Analysis Between GNG7 Expression and Immune Markers

To further investigate the relationships between GNG7 and immune infiltrating cells, we used TIMER and GEPIA database to analyze the correlations between GNG7 expression and immune cell markers of diverse immune cells in COAD and READ. These immune cell markers were used to characterize immune cells, including B cells, CD8^+^ T cells, T cells (general), M1/M2 macrophages, tumor-associated macrophages (TAMs), monocytes, NK cells, neutrophils and DCs. We also analyzed different functional T cells, such as Th1 cells, Th2 cells, Tfh cells, Th17 cells, and Tregs, as well as exhausted T cells. The results revealed that GNG7 was significantly correlated with most immune marker sets of multiple immune cells and different T cells in COAD and READ (Table 2). In contrast, only 13 immune cell gene markers in LIHC were significantly correlated with the GNG7 expression.

**TABLE 2:** Correlation analysis between GNG7 and relate genes and markers of immune infiltration cells in TIMER.

Description	Gene markers	COAD				READ				LIHC			
		None		Purity		None		Purity		None		Purity	
		Cor	P	Cor	P	Cor	P	Cor	P	Cor	P	Cor	P
CD8^+^ T cell	CD8A	0.361	***	0.238	***	0.425	***	0.33	***	0.01	0.849	0.063	0.243
	CD8B	0.207	***	0.131	**	0.182	*	0.158	0.0638	−0.046	0.38	−0.008	0.877
T cell (general)	CD3D	0.362	***	0.229	***	0.332	***	0.215	*	−0.135	**	−0.105	0.051
	CD3E	0.459	***	0.348	***	0.454	***	0.366	***	−0.034	0.516	0.011	0.839
	CD2	0.418	***	0.292	***	0.412	***	0.307	***	−0.034	0.51	0.007	0.903
B cell	CD19	0.499	***	0.428	***	0.36	***	0.323	***	0.022	0.669	0.043	0.427
	CD79A	0.579	***	0.505	***	0.448	***	0.355	***	0.02	0.708	0.058	0.283
Monocyte	CD86	0.435	***	0.294	***	0.424	***	0.339	***	−0.059	0.253	−0.035	0.522
	CD115(CSF1R)	0.517	***	0.401	***	0.568	***	0.532	***	0.027	0.599	0.054	0.319
TAM	CCL2	0.407	***	0.294	***	0.493	***	0.414	***	0.082	0.114	0.127	*
	CD68	0.338	***	0.219	***	0.445	***	0.424	***	−0.094	0.071	−0.067	0.216
	IL10	0.328	***	0.226	***	0.256	***	0.193	*	−0.107	*	−0.101	0.06
M1 Macrophage	INOS(NOS2)	0.002	0.961	−0.047	0.346	0.025	0.751	0.046	0.592	0.161	**	0.158	**
	IRF5	0.26	***	0.233	***	0.2	*	0.209	*	0.019	0.709	0.01	0.859
	COX2(PTGS2)	0.193	***	0.134	**	0.2	**	0.107	0.208	0.02	0.704	0.047	0.384
M2 Macrophage	CD163	0.455	***	0.33	***	0.508	***	0.435	***	0.04	0.44	0.069	0.198
	VSIG4	0.427	***	0.304	***	0.45	***	0.375	***	0.004	0.937	0.034	0.533
	MS4A4A	0.414	***	0.279	***	0.494	***	0.408	***	0.025	0.636	0.066	0.224
Neutrophils	CD66b (CEACAM8)	−0.117	*	−0.072	0.145	−0.148	0.0563	−0.072	0.399	−0.068	0.194	−0.06	0.266
	CD11b (ITGAM)	0.427	***	0.322	***	0.482	***	0.392	***	−0.07	0.176	−0.084	0.117
	CCR7	0.495	***	0.399	***	0.365	***	0.317	***	0.082	0.114	0.118	*
Natural killer cell	KIR2DL1	0.206	***	0.134	**	0.141	0.0707	0.077	0.365	0.03	0.568	0.031	0.569
	KIR2DL3	0.143	**	0.038	0.443	0.198	*	0.144	0.0905	0.118	*	0.141	**
	KIR2DL4	0.185	***	0.079	0.114	0.196	*	0.091	0.287	0.006	0.905	0.029	0.592
	KIR3DL1	0.168	***	0.053	0.290	0.148	0.0574	0.064	0.452	0.16	**	0.183	***
	KIR3DL2	0.26	***	0.172	***	0.304	***	0.232	**	0.074	0.157	0.125	*
	KIR3DL3	0.069	0.138	0.018	0.713	0.022	0.78	0.012	0.893	0.003	0.954	0.009	0.869
	KIR2DS4	0.137	**	0.082	0.097	0.123	0.114	0.057	0.502	0.128	*	0.139	**
Dendritic cell	HLA−DPB1	0.44	***	0.298	***	0.453	***	0.372	***	0.053	0.306	0.087	0.105
	HLA−DQB1	0.308	***	0.181	***	0.18	*	0.09	0.29	−0.039	0.449	−0.011	0.834
	HLA−DRA	0.355	***	0.204	***	0.366	***	0.244	**	0.034	0.516	0.064	0.239
	HLA−DPA1	0.424	***	0.283	***	0.456	***	0.36	***	0.058	0.267	0.089	0.101
	BDCA−1(CD1C)	0.466	***	0.381	***	0.538	***	0.468	***	0.018	0.726	0.034	0.534
	BDCA−4(NRP1)	0.446	***	0.323	***	0.571	***	0.505	***	0.139	**	0.152	**
	CD11c (ITGAX)	0.445	***	0.315	***	0.456	***	0.385	***	−0.138	**	−0.143	**
Th1	T−bet (TBX21)	0.374	***	0.261	***	0.435	***	0.367	***	0.063	0.229	0.092	0.088
	STAT4	0.417	***	0.303	***	0.422	***	0.334	***	−0.044	0.397	−0.026	0.628
	STAT1	0.233	***	0.122	*	0.259	***	0.172	*	0.027	0.607	0.043	0.422
	IFN−γ (IFNG)	0.126	**	0.023	0.645	0.148	0.0576	0.04	0.638	0.002	0.964	0.034	0.525
	TNF−α (TNF)	0.229	***	0.146	**	0.333	***	0.272	**	−0.037	0.483	−0.036	0.501
Th2	GATA3	0.488	***	0.397	***	0.465	***	0.399	***	0.011	0.834	0.043	0.421
	STAT6	0.175	***	0.195	***	0.224	**	0.309	***	0.268	***	0.259	***
	STAT5A	0.3	***	0.242	***	0.163	*	0.159	0.0609	0.171	***	0.198	***
	IL13	0.155	***	0.07	0.157	0.105	0.179	−0.021	0.807	−0.025	0.632	−0.056	0.298
Tfh	BCL6	0.41	***	0.311	***	0.44	***	0.439	***	0.043	0.414	0.029	0.591
	IL21	0.133	**	0.079	0.113	0.078	0.32	0.082	0.34	0.032	0.54	0.032	0.559
Th17	STAT3	0.312	***	0.239	***	0.398	***	0.383	***	0.037	0.477	0.042	0.435
	IL17A	−0.063	0.179	−0.08	0.106	−0.078	0.316	−0.062	0.472	−0.032	0.541	−0.006	0.91
Treg	FOXP3	0.45	***	0.343	***	0.479	***	0.433	***	0.025	0.638	0.017	0.755
	CCR8	0.412	***	0.317	***	0.475	***	0.407	***	−0.053	0.308	−0.05	0.358
	STAT5B	0.314	***	0.32	***	0.442	***	0.443	***	0.225	***	0.207	***
	TGFβ(TGFB1)	0.504	***	0.386	***	0.397	***	0.301	***	−0.073	0.158	−0.053	0.324
T cell exhaustion	PD−1(PDCD1)	0.366	***	0.26	***	0.382	***	0.297	***	−0.067	0.199	−0.031	0.566
	CTLA4	0.369	***	0.253	***	0.37	***	0.294	***	−0.152	**	−0.127	*
	LAG3	0.351	***	0.235	***	0.363	***	0.303	***	−0.073	0.16	−0.044	0.412
	TIM−3(HAVCR2)	0.42	***	0.281	***	0.398	***	0.313	***	−0.093	0.072	−0.078	0.148
	GZMB	0.02	0.674	−0.033	0.511	0.134	0.0848	0.077	0.37	0.041	0.433	0.067	0.212

*p* < 0.05 were considered to be statistically significant (*p < 0.05, **p < 0.01, ***p < 0.001).

Interestingly, we found that compared with COAD, the expression of GNG7 in READ is associated with more immune infiltrating cell markers and the correlation coefficient is higher. As shown in Table 2, among these TILs, B cells, TAM, M2 macrophages, dendritic cells and Treg cells displayed stronger correlation with GNG7 expression than others. Therefore, we further explored the correlation between GNG7 expression in COAD, READ and these gene markers by using GEPIA database. The analysis results showed that the degree of correlation is basically the same as the results we obtained in TIMER Table 3.

**TABLE 3:** Correlation analysis between GNG7 and relate genes and markers of immune infiltration cells in GEPIA.

Description	Gene markers	COAD				READ			
		Tumor		Normal		Tumor		Normal	
		R	P	R	P	R	P	R	P
T cell (general)	CD3E					0.5	***	0.56	0.093
B cell	CD19	0.49	***	0.3	0.056				
	CD79A	0.58	***	0.4	*	0.48	***	0.58	0.077
Monocyte	CD115(CSF1R)	0.52	***	0.35	0.027	0.68	***	0.6	0.069
TAM	CCL2					0.61	***	0.71	0.021
	CD68					0.51	***	−0.17	0.64
M2 Macrophage	CD163					0.52	***	0.62	0.056
	VSIG4					0.55	***	−0.018	0.96
	MS4A4A					0.56	***	0.03	0.93
Neutrophils	CD11b (ITGAM)					0.54	***	0.68	0.03
	CCR7	0.5	***	0.19	0.23				
Dendritic cell	HLA−DPB1					0.6	***	0.079	0.83
	HLA−DPA1					0.57	***	0.44	0.21
	BDCA−1(CD1C)	0.52	***	0.2	0.2	0.55	***	0.63	0.05
	BDCA−4(NRP1)					0.65	***	0.84	*
	CD11c (ITGAX)					0.51	***	0.66	0.037
Th1	T−bet (TBX21)					0.55	***	0.63	0.053
Th2	GATA3	0.55	***	0.23	0.15	0.55	***	0.63	0.05
Tfh	BCL6					0.58	***	0.85	*
Th17	STAT3					0.43	***	0.32	0.36
Treg	FOXP3					0.5	***	0.4	0.26
	CCR8					0.45	***	0.44	0.21
	STAT5B					0.43	**	0.94	**
	TGFβ(TGFB1)	0.49	***	0.61	***				

Notes: According to the analysis results in TIMER, all gene markers with correlation coefficients greater than 0.35 after adjustment of tumor purity were verified again in GEPIA, and all the verification results are listed in this table. (*p < 0.01; **p < 0.001; ***p < 0.0001).

Consequently, we have reason to believe that GNG7 is closely correlated to immune cell infiltration in CRC, which guides us to speculate that GNG7 plays an important role in the tumor microenvironment and immune response of CRC.

## Discussion

Colorectal cancer is a kind of cancer with a high level of morbidity and mortality worldwide and its clinical diagnosis and treatment progress has always been focused on (Arnold et al., 2017). Moreover, due to the lack of effective early detection methods, combined with its low penetration, many patients have already reached advanced stage at the time they received clinical diagnosis (Dekker et al., 2019). Therefore, the discovery of new, relatively convenient, and non-invasive detection markers will help improve the early screening rate of CRC, thereby increasing the chance of patients being treated and improving the prognosis of patients.

In recent years, with the progress of tumor molecular biology research, more and more new and effective markers have been discovered, for example, Ritwik Patra’s research found that COL11A1 gene mutation profoundly affects the occurrence and development of colorectal cancer, and confirmed the qualification of COL11A1 gene as a potential biomarker for colorectal cancer prognosis (Patra et al., 2021). In addition, it is reported that the use of combined detection of SDC2 and TFPI2 methylation as a new biomarker for a non-invasive tool of CRC will help improve the current dilemma of low early screening rates for CRC (Zhang et al., 2021). Meanwhile, as a cell cycle-related protein, SEPT9 has been confirmed to be involved in tumorigenesis and development in a variety of malignant tumors, and the methylation detection of SERT9 gene in peripheral blood has high sensitivity and specificity for the diagnosis of CRC, and can be used in early CRC screening, it may be a useful biomarker for the prognosis of CRC (Mostowy and Cossart 2012; Sun et al., 2019; Yang et al., 2019).

Immune cells are an important part of tumor stroma. Accumulating evidences suggests that innate immune cells (including macrophages, neutrophils, dendritic cells, NK cells, innate lymphocytes and myeloid-derived suppressor cells) as well as adaptive immune cells (T cells and B cells) participate in the formation of the tumor microenvironment (TME). The crosstalk between cancer cells and proximal immune cells will eventually create an environment that fosters tumor growth and metastasis (Gajewski et al., 2013; Hinshaw and Shevde 2019). Moreover, the high level of tumor immune infiltration may be related to poor prognosis in CRC (Guo et al., 2020).

At present, with the in-depth understanding of the mechanism of tumor immune infiltration, the tumor immunotherapy has also been rapidly developed. In particular, tumor immunotherapy has yielded significant effects in a variety of cancer types (Rosenberg et al., 2019; Brewer et al., 2020; Farid and Liu 2020).

However, the progress of immunotherapy for colorectal cancer is still relatively limited. The most important reason is that most CRCs are microsatellite stable, but it is disappointing that currently immunotherapy only produces a good response in a small subset of CRCs that exhibit microsatellite instability (MSI), and the difference in the sensitivity of the two types of CRC to immunotherapy is mainly related to the immune microenvironment at the primary site of the tumor (Mlecnik et al., 2016). Therefore, discovering new immune-related markers and therapeutic targets become the main content of research on CRC immunotherapy.

This study aims to predict the role of GNG7 in CRC by studying the relationship between the expression level of GNG7 and the infiltration level of different immune cells in colorectal cancer and the prognosis of patients with CRC through multi-channel bioinformatics data mining and analysis.

GNG7 is located on chromosome 19 and is a member of the guanine nucleotide-binding proteins (G proteins) family which is involved as a modulator or transducer in various transmembrane signaling systems (Shibata et al., 1998; Hartmann et al., 2012). Previous studies have reported that the loss of GNG7 is related to the tumor volume of esophageal cancer and affects tumor invasiveness, which may attributed to loss of heterozygosity (LOH) (Ohta et al., 2008). Zheng H et al.'s study on LUAD found that GNG7 can exert a tumor suppressor effect in LUAD cells by inhibiting E2F1, moreover, low GNG7 expression is significantly associated with a poorer LUAD prognosis and a higher tumor grade (Zheng et al., 2021). However, the role of GNG7 in colorectal cancer remains to be elucidated. In order to acquire more detailed information about the potential role and biological mechanism of GNG7 expression in CRC, we conducted a stepwise bioinformatics analysis. First, we performed analyses in Oncomine database and TIMER database and found that compared to normal tissues, GNG7 expression in CRC was significantly down-regulated. In addition, the findings from UALCAN indicated that GNG7 expression is associated with tumor histology, stage, lymph node metastasis in colorectal cancer which means that GNG7 may play an important role in the occurrence and progression of colorectal cancer. In addition, we further carried out the qRT-PCR experiment of clinical colorectal tissue samples, and the results are consistent with the results in the database as well. Meanwhile, it is worth noting that analysis of the Oncomine and TIMER databases revealed that the expression levels of GNG7 in various cancers were different relative to normal tissues, however, GNG7 was down-regulated in some cancers and up-regulated in others. Unfortunately, The specific mechanism causing this result has not yet been fully elucidated. We speculate that this may be related to differences in cancer cell tissue types, or that the changes in signaling pathways vary in different cancers, which may also lead to increased or decline expression of downstream proteins. which needs to be determined by further basic experimental research in the future.

Cancer cells are distinguished from normal cells by two major characteristics: immortal proliferation and decreased apoptosis (Hanahan and Weinberg 2011). It is reported that GNG7 has the ability to induce apoptosis and arrest the cell cycle, which may be related to the reduction of actin polymers in cells. In addition, GNG7 induces autophagy by inhibiting the MTOR pathway, thereby limiting the number of cells, reducing the proliferation ability to a certain extent, and playing the role of tumor suppressor genes (Liu et al., 2016; Lai and Ding 2019).

Our survival analysis results in the database showed that GNG7 expression is correlated to the prognosis of different cancers, including CRC. Low GNG7 level means poor colorectal cancer DSS and OS. Therefore, we speculated that GNG7 exhibits significance as a prognostic marker in CRC.

Another important part of this study is to explore the correlation between the expression of GNG7 and the level of infiltration of different immune cells as well as immunoregulators in cancer, especially in colorectal cancer. Our analysis showed that the relationship between GNG7 expression and the level of CD4^+^ T cell infiltration in CRC is moderate to strong. Simultaneously, there is a positive correlation between GNG7 expression and the infiltration level of B cells, macrophages, neutrophils and dendritic cells. In addition, the GNG7 expression has a significant correlation with numerous immunoregulators as well as some immune cell marker genes in COAD and READ, which implies that GNG7 may be involved in the tumor immune process of COAD and READ, and may play a significant role in tumor-related immune activation or suppression. Hence, we hypothesized that GNG7 is a potential marker for predicting tumor immune microenvironment homeostasis.

Macrophages belong to a differentiated subtype of immune cells, which can be divided into M1 macrophages and M2 macrophages (Jayasingam et al., 2020). They play an important role in the development, homeostasis and immune response of organism (Wynn et al., 2013). Increased macrophage infiltration in colorectal cancer tissues was significantly associated with increased chemoresistance and poor prognosis (Yin et al., 2017).

It has now been fully confirmed that a large number of Treg cell infiltrations in tumor tissues of various cancers are ubiquitous, and their abundant presence often indicates a poor clinical prognosis. Among the various mechanisms of immunological self-tolerance, endogenous Treg cell-mediated immunosuppression is essential (Tanaka and Sakaguchi 2017). Dendritic cell is a quintessential APC of the immune system, responsible for combining innate immunity and adaptive immunity, including activation of anti-tumor T cells (Broz et al., 2014; Gardner et al., 2020). And dendritic cell immunotherapy has received good feedback in animal experiments, so it is regarded as a new cancer therapy which is worth looking forward to (Cranmer et al., 2004). Additionally, DC can assist tumor metastasis by increasing Treg cells and reducing the toxicity of CD8^+^ T cells (Sawant et al., 2012). These underlying mechanisms may be the basis for GNG7 to recruit and regulate immune cells. This provides a perspective for us to understand how the low expression of GNG7 affects the prognosis of CRC by regulating immune infiltration.

Therefore, we can conclude that GNG7 may be a potential biomarker and is related to the immune infiltration of colorectal cancer, which means that GNG7 can be used as a potential target for regulating anti-tumor immunotherapy. This study provided insights for further research on immunotherapy of CRC. Nevertheless, this study existed limitations, the data of this study is mainly based on data obtained in open access databases, lacking multi-center and large sample data support. In addition, the relevant mechanism of GNG7 in recruiting and regulating immune cells as well as immunoregulators needs to be further explored at the cellular and molecular level and in animal experiments in the future.

## Conclusion

Our study confirmed that GNG7 expression is down-regulated in colorectal cancer, and GNG7 expression is related to the prognosis of colorectal cancer. moreover, the correlation between GNG7 gene and multiple immune cell infiltration levels, immunomodulatory molecules, and immune cell related genes in colorectal cancer revealed that GNG7 has a potential role in regulating immune cell function in colorectal cancer, which may provide a new insight to individualized treatment for CRC. In addition, further research should aim to elucidate the molecular mechanisms that may help determine the immune target of CRC.

## Data Availability

The datasets presented in this study can be found in online repositories. The names of the repository/repositories and accession number(s) can be found in the article/Supplementary Material.

## Glossary

ccRCC: clear cell renal cell carcinoma
CNS: central nervous system
COAD: colon adenocarcinoma
CRC: colorectal cancer
dcs: dendritic cells
DMFS: distant metastasis free survival
DSS: disease specific survival
GNG7: G protein subunit gamma 7
HR: hazard ratio
ICIS: immune checkpoint inhibitors
LIHC: liver hepatocellular carcinoma
MHC molecule: major histocompatibility complex molecule
NK cells: natural killer cells
NK T cells: natural killer T cells
OS: overall survival
READ: rectum adenocarcinoma
RFS: relapse-free survival
tams: tumor-associated macrophages
TCGA: the cancer genome atlas
tems: Tie2-expressing monocytes
tfh: T follicular helper cell
th 1 cells: type I helper T cells
th 2 cells: type II helper T cells
Th17 cells: type 17 T helper cells
TIIC: tumor infiltrating immune cell
tils: tumor infiltrating lymphocytes
TIMER: tumor immune estimation resource
TME: tumor microenvironment
treg: regulatory T cells
