# Supplementary material for: The Prognostic Value of GNG7 in Colorectal Cancer and Its Relationship With Immune Infiltration
Source: Front Genet. 2022 Feb 23;13:833013. doi: 10.3389/fgene.2022.833013 (PMC8906903; doi:10.3389/fgene.2022.833013)
Supplement: Supplementary file 1 [file Table3.doc]

**Supplementary Table 3**. The correlation between GNG7 expression and main immunoregulators in COAD and READ (TISIDB)

| Immunoregulators | COAD | | Immunoregulators | READ | |
| --- | --- | --- | --- | --- | --- |
|  | Cor | p |  | Cor | p |
| **Immunoinhibitors** |  |  | **Immunoinhibitors** |  |  |
| BTLA | 0.451 | <2.2e-16 | CSF1R | 0.513 | <2.2e-16 |
| CSF1R | 0.444 | <2.2e-16 | ADORA2A | 0.469 | 2.27e-10 |
| ADORA2A | 0.428 | <2.2e-16 | KDR | 0.338 | 9.21e-06 |
| **Immunostimulators** |  |  | **Immunostimulators** |  |  |
| CD27 | 0.517 | <2.2e-16 | CXCL12 | 0.46 | 6.26e-10 |
| TNFRSF13B | 0.515 | <2.2e-16 | CD28 | 0.451 | 1.44e-09 |
| TNFRSF17 | 0.45 | <2.2e-16 | CXCR4 | 0.437 | 4.95e-09 |
| **major histocompatibility complex (MHC) molecules** |  |  | **major histocompatibility complex (MHC) molecules** |  |  |
| HLA-DOA | 0.412 | <2.2e-16 | HLA-DOA | 0.468 | 2.64e-10 |
| HLA-DPB1 | 0.39 | <2.2e-16 | HLA-DPB1 | 0.404 | 8.15e-08 |
| HLA-DQA1 | 0.384 | <2.2e-16 | HLA-DPA1 | 0.375 | 7.01e-07 |
