# Supplementary material for: The Prognostic Value of GNG7 in Colorectal Cancer and Its Relationship With Immune Infiltration
Source: Front Genet. 2022 Feb 23;13:833013. doi: 10.3389/fgene.2022.833013 (PMC8906903; doi:10.3389/fgene.2022.833013)
Supplement: Supplementary file 2 [file Table1.doc]

**Supplementary Table 1**. GNG7 expression in cancers versus normal tissue in Oncomine database

| Cancer | Cancer subtype | P value | Fold change | Rank (%) | Sample | Reference (PMID) |
| --- | --- | --- | --- | --- | --- | --- |
| Bladder | Infiltrating Bladder Urothelial Carcinoma | 2.26E-13 | -2.525 | 1% | 130 | 20421545 |
|  | Superficial Bladder Cancer | 5.14E-15 | -2.375 | 2% | 194 | 20421545 |
|  |  |  |  |  |  |  |
| Brain and CNS | Anaplastic Oligodendroglioma | 0.001 | -3.286 | 5% | 7 | 16204036 |
|  | Oligodendroglioma | 0.002 | -2.631 | 6% | 9 | 16204036 |
|  |  |  |  |  |  |  |
| Breast | Ductal Breast Carcinoma in Situ Stroma | 8.26E-07 | -2.169 | 1% | 25 | 19187537 |
|  | Invasive Breast Carcinoma | 8.99E-19 | -3.083 | 4% | 137 | TCGA |
|  | Invasive Ductal Breast Carcinoma | 2.66E-25 | -3.221 | 6% | 450 | TCGA |
|  | Mucinous Breast Carcinoma | 2.00E-03 | -2.386 | 7% | 65 | TCGA |
|  |  |  |  |  |  |  |
| Colorectal | Rectal Adenocarcinoma | 1.33E-30 | -6.254 | 1% | 82 | TCGA |
|  | Colon Adenocarcinoma | 1.41E-32 | -6.155 | 1% | 123 | TCGA |
|  | Colon Mucinous Adenocarcinoma | 4.60E-18 | -4.151 | 1% | 44 | TCGA |
|  | Rectosigmoid Adenocarcinoma | 5.66E-13 | -5.782 | 1% | 25 | TCGA |
|  | Rectal Mucinous Adenocarcinoma | 8.49E-08 | -4.298 | 2% | 28 | TCGA |
|  | Cecum Adenocarcinoma | 5.38E-14 | -5.064 | 2% | 44 | TCGA |
|  | Rectal Adenocarcinoma | 9.87E-37 | -3.342 | 1% | 130 | 20725992 |
|  | Colon Adenoma | 4.85E-11 | -2.034 | 2% | 57 | 18171984 |
|  | Colon Carcinoma | 1.57E-07 | -2.171 | 4% | 15 | 20957034 |
|  | Colorectal Carcinoma | 1.15E-09 | -2.302 | 5% | 82 | 20143136 |
|  |  |  |  |  |  |  |
| Gastric | Diffuse Gastric Adenocarcinoma | 1.81E-09 | -2.49 | 1% | 50 | 21447720 |
|  | Gastric Intestinal Type Adenocarcinoma | 7.82E-07 | -2.091 | 1% | 39 | 21447720 |
|  | Gastric Mixed Adenocarcinoma | 5.52E-06 | -2.788 | 2% | 29 | 21447720 |
|  | Gastric Adenocarcinoma | 6.00E-03 | -2.531 | 5% | 23 | 21447720 |
|  |  |  |  |  |  |  |
| Head and Neck | Salivary Gland Adenoid Cystic Carcinoma | 1.67E-09 | -4.015 | 1% | 22 | 12368205 |
|  |  |  |  |  |  |  |
| Kidney | Renal Oncocytoma | 3.00E-03 | 3.944 | 10% | 9 | 19445733 |
|  |  |  |  |  |  |  |
| Leukemia | B-Cell Acute Lymphoblastic Leukemia | 2.29E-75 | 4.863 | 1% | 221 | 20406941 |
|  | B-Cell Childhood Acute Lymphoblastic Leukemia | 6.79E-108 | 3.671 | 1% | 433 | 20406941 |
|  | Chronic Lymphocytic Leukemia | 9.62E-71 | 2.356 | 2% | 522 | 20406941 |
|  | B-Cell Acute Lymphoblastic Leukemia | 2.86E-58 | 2.673 | 2% | 172 | 20406941 |
|  | T-Cell Acute Lymphoblastic Leukemia | 1.06E-07 | -9.404 | 4% | 15 | 17410184 |
|  | Hairy Cell Leukemia | 2.61E-06 | -2.511 | 7% | 41 | 15778709 |
|  |  |  |  |  |  |  |
| Lung | Lung Adenocarcinoma | 1.63E-04 | 2.018 | 7% | 57 | 17540040 |
|  | Small Cell Lung Carcinoma | 9.18E-04 | -2.508 | 24% | 23 | 11707567 |
|  |  |  |  |  |  |  |
| Lymphoma | Diffuse Large B-Cell Lymphoma | 2.23E-12 | -3.462 | 1% | 57 | 15778709 |
|  | Centroblastic Lymphoma | 4.70E-11 | -3.403 | 3% | 53 | 15778709 |
|  | Follicular Lymphoma | 0.002 | -2.63 | 4% | 31 | 15778709 |
|  | Diffuse Large B-Cell Lymphoma | 7.29E-12 | -2.008 | 1% | 36 | 18794340 |
|  | Activated B-Cell-Like Diffuse Large B-Cell Lymphoma | 3.47E-14 | -2.965 | 1% | 37 | 19412164 |
|  | Diffuse Large B-Cell Lymphoma | 1.49E-10 | -2.35 | 9% | 64 | 19412164 |
|  |  |  |  |  |  |  |
| Ovarian | Ovarian Serous Adenocarcinoma | 5.61E-07 | -2.31 | 10% | 53 | 19486012 |
|  |  |  |  |  |  |  |
| Pancreatic | Pancreatic Carcinoma | 6.93E-09 | -2.051 | 1% | 52 | 19732725 |
|  |  |  |  |  |  |  |
| Other | Skin Squamous Cell Carcinoma | 0.002 | -2.506 | 3% | 15 | 18442402 |
