# Supplementary material for: The Prognostic Value of GNG7 in Colorectal Cancer and Its Relationship With Immune Infiltration
Source: Front Genet. 2022 Feb 23;13:833013. doi: 10.3389/fgene.2022.833013 (PMC8906903; doi:10.3389/fgene.2022.833013)
Supplement: Supplementary file 3 [file Table2.doc]

**Supplementary Table 2**. The clinical characteristics of experimental samples

| Sample | Race | Age | Gender | Organ/Anatomic site | Pathology Diagnosis | Differentiated Grade | TNM stage |
| --- | --- | --- | --- | --- | --- | --- | --- |
| 1 | han | 88 | male | sigmoid | adenocarcinoma | middle | T3N0Mx |
| 2 | han | 45 | male | sigmoid | adenocarcinoma | middle | T3N0Mx |
| 3 | han | 65 | female | ileocecal junction | adenocarcinoma | middle | T2N0Mx |
| 4 | han | 88 | female | right colon | adenocarcinoma | middle | T3N0Mx |
| 5 | han | 69 | male | right colon | adenocarcinoma | middle | T3N0Mx |
| 6 | han | 59 | male | sigmoid | adenocarcinoma | middle | T3N0Mx |
| 7 | han | 72 | female | right colon | adenocarcinoma | middle | T3N1Mx |
| 8 | han | 64 | male | right colon | adenocarcinoma | middle | T3N1M1 |
| 9 | han | 46 | male | right colon | adenocarcinoma | middle | T2N0Mx |
| 10 | han | 54 | male | left colon | adenocarcinoma | middle | T3N0Mx |
| 11 | han | 67 | male | right colon | adenocarcinoma | middle | T3N0Mx |
| 12 | han | 70 | male | rectum | adenocarcinoma | middle | T3N2Mx |
| 13 | han | 72 | male | sigmoid | adenocarcinoma | middle | T3N1Mx |
| 14 | han | 58 | female | rectum | adenocarcinoma | middle | T3N0Mx |
| 15 | han | 64 | female | rectum | adenocarcinoma | middle | T3N0Mx |
| 16 | han | 40 | female | sigmoid | adenocarcinoma | middle | T3N0Mx |
| 17 | han | 71 | male | rectum | adenocarcinoma | middle | T3N0Mx |
| 18 | han | 65 | male | sigmoid | adenocarcinoma | middle | T3N1Mx |
| 19 | han | 71 | male | left colon | mucous adenocarcinoma | poorly | T4N2Mx |
| 20 | han | 62 | female | left colon | adenocarcinoma | middle | T2N0Mx |

note: Tissue specimen collection time is from June 10, 2021 to July 20, 2021
